# Extreme endurance flights by landbirds crossing the Pacific Ocean: ecological corridor rather than barrier?

**DOI:** 10.1098/rspb.2008.1142

**Published:** 2008-10-29

**Authors:** Robert E. Gill, T. Lee Tibbitts, David C. Douglas, Colleen M. Handel, Daniel M. Mulcahy, Jon C. Gottschalck, Nils Warnock, Brian J. McCaffery, Philip F. Battley, Theunis Piersma

**Affiliations:** 1 USGS Alaska Science Center4210 University Drive, Anchorage, AK 99508, USA; 2 USGS Alaska Science Center3100 National Park Road, Juneau, AK 99801, USA; 3 Climate Prediction Center, NOAA5200 Auth Road, Camp Springs, MD 20746, USA; 4 PRBO Conservation Science3820 Cypress Drive, Petaluma, CA 94954, USA; 5 US Fish and Wildlife Service, Yukon Delta National Wildlife RefugePO Box 346, Bethel, AK 99559, USA; 6 School of Biological Sciences, University of Auckland1142 Auckland, New Zealand; 7 Royal Netherlands Institute for Sea Research (NIOZ), Texel, and Animal Ecology Group, Centre for Ecological and Evolutionary Studies, University of Groningen9750 AA Haren, The Netherlands

**Keywords:** bar-tailed godwit, satellite telemetry, endurance exercise, migration, climate change, weather

## Abstract

Mountain ranges, deserts, ice fields and oceans generally act as barriers to the movement of land-dependent animals, often profoundly shaping migration routes. We used satellite telemetry to track the southward flights of bar-tailed godwits (*Limosa lapponica baueri*), shorebirds whose breeding and non-breeding areas are separated by the vast central Pacific Ocean. Seven females with surgically implanted transmitters flew non-stop 8117–11 680 km (10 153±1043 s.d.) directly across the Pacific Ocean; two males with external transmitters flew non-stop along the same corridor for 7008–7390 km. Flight duration ranged from 6.0 to 9.4 days (7.8±1.3 s.d.) for birds with implants and 5.0 to 6.6 days for birds with externally attached transmitters. These extraordinary non-stop flights establish new extremes for avian flight performance, have profound implications for understanding the physiological capabilities of vertebrates and how birds navigate, and challenge current physiological paradigms on topics such as sleep, dehydration and phenotypic flexibility. Predicted changes in climatic systems may affect survival rates if weather conditions at their departure hub or along the migration corridor should change. We propose that this transoceanic route may function as an ecological corridor rather than a barrier, providing a wind-assisted passage relatively free of pathogens and predators.

## 1. Introduction

The evolution of migration pathways on the global landscape has been constrained by prominent ecological barriers, namely oceans, mountain ranges, deserts and ice fields. These features act as barriers to migration because they are largely absent of the food and water necessary to replenish energy stores for continued migratory flight, and they offer inhospitable environments for stopping (e.g. severe heat, cold or turbulence). Some are so large as to preclude many birds from carrying sufficient stores to cross them. Some of the most important insights into the limitations of avian flight performance and migration ecology have come from the relatively few species that have evolved strategies to cross such features routinely (Berthold *et al.* 2003; Alerstam *et al.* 2007; Schmaljohann *et al.* 2007; Newton 2008). Until now, the longest documented non-stop flight of any landbird has been a crossing of the western Pacific Ocean by far eastern curlews (*Numenius madagascariensis*) flying for 3–5 days between eastern Australia and China, a distance of approximately 6500 km with an overwater leg of approximately 4500 km (Driscoll & Ueta 2002). In a previous study, Gill *et al.* (2005) presented several lines of evidence that suggested that some, if not most, bar-tailed godwits (*Limosa lapponica baueri*) make a direct, non-stop flight across the central Pacific Ocean from Alaska to New Zealand and eastern Australia, covering a minimum distance of approximately 10 000 km. This far surpassed previous maximum flight range estimates for birds with flapping flight (Williams & Williams 1999; Pennycuick & Battley 2003). Indirect support for the hypothesis came from the analyses of temporal and spatial distribution of sightings of leg-banded birds along plausible migration routes, the timing of departures and arrivals at migration termini, flight performance modelling (Pennycuick & Battley 2003; Gill *et al.* 2005) and analyses of meteorological conditions. In this study, we directly tested the hypothesis of a non-stop southward flight across the central Pacific Ocean by tracking the flight paths of nine individual godwits that we implanted with miniaturized satellite transmitters.

## 2. Material and methods

### (a) Capturing and instrumenting birds

We tracked the southward flights of godwits from the breeding grounds using satellite transmitters (PTTs; Microwave Telemetry, Inc., Columbia, MD, USA). During June 2006, we captured four females and three males on the breeding grounds in western Alaska (61.4° N, 165.5° W) to assess southward migration. During February 2007, we captured eight males and eight females on the non-breeding areas in New Zealand (Golden Bay, 40.4° S, 172.7° E; Miranda, 37.2° S, 175.3° E) specifically to assess northward migration; however, battery life of these transmitters exceeded expectations, allowing us to continue to track some of these during southward migration. We used two models of PTTs: a 26 g battery-powered model was surgically implanted in the coelomic cavity (Korschgen *et al.* 1996; Mulcahy & Esler 1999) of female godwits and a 10.5 g solar-powered model was attached to the lower backs of male godwits using a leg-loop harness. PTTs fitted to nesting godwits ranged between 6.4 and 7.5% of body mass for the implantable model and between 4.3 and 4.7% for the externally attached model. Male godwits are structurally smaller and lighter (15–28%) than females (McCaffery & Gill 2001) and could only carry the smaller externally attached models. PTTs fitted to New Zealand birds ranged from 4.7 to 5.7% and from 2.9 to 3.2% of live body mass for implantable and solar-powered models, respectively. Body mass of godwits at the onset of migration is probably double that during the periods of PTT attachment (cf. Piersma & Gill 1998; McCaffery & Gill 2001; Battley & Piersma 2005); thus the ratio of PTT mass to body mass was considerably reduced at the time of departure. To reduce potential wind drag, we contoured antennas of both PTT models to the birds' in-flight horizontal plane. To facilitate subsequent resightings, all the birds were marked with uniquely coded leg flags.

Among the 23 transmitters deployed, nine functioned properly during southward flights, comprising those on three females and two males instrumented in Alaska and four females instrumented in New Zealand. Among five other females with implanted PTTs, one did not migrate north to breeding grounds and four had batteries expire before southward migration had begun. Nine external PTTs fitted to males stopped reporting before southward migration, although several of these birds were subsequently resighted. Some males were found to have shed their transmitters, probably owing to migration-related changes in body mass and shape. Other transmitters may have ceased to function when they shifted into a position that did not allow solar cells to recharge.

### (b) Data transmission and acquisition

During the southward migration period in 2006, implantable PTTs were programmed to transmit signals for 8 hours and then rest for 24 hours to extend battery life; in 2007 they were programmed to transmit for 6 hours and rest for 36 hours. Solar-powered PTTs were permanently scheduled to transmit for 10 hours, separated by 48 hours recharging intervals. Argos data collection system receivers aboard NOAA polar-orbiting satellites collected the PTT transmissions. CLS America, Inc. (http://www.clsamerica.com) derived location estimates based on satellite overpass geometry and Doppler shifts in the PTT uplink frequency (401.65 MHz). We filtered locations based on location accuracy, spatial redundancy and a systematic algorithm to remove improbable locations. We calculated tracking distances using the standard orthodrome (great circle) formula. Here, we distinguish the associated track speed from true flight speed owing to the uncertainties introduced by discontinuous tracking and imprecise locations. Furthermore, we have not attempted in this paper to decompose track speeds into their respective ground-speed and wind-speed vector components. Onset and termination points of the migratory flights were assumed to be the last and first terrestrial (or last open ocean) locations recorded in Alaska and the South Pacific, respectively. Since tracking relocations were intermittent due to PTT duty cycles and satellite overpass frequency, we estimated the departure times by extrapolating the net tracking velocity during the first in-flight duty cycle from the first in-flight location back to the departure location. Arrival times were analogously estimated using velocity data from the last in-flight duty cycle extrapolated from the last in-flight location forward to the arrival location.

### (c) Filtering of location data

CLS America, Inc., calculated and disseminated the locations of godwits with an index of accuracy. Standard location classes (LC 1, 2 or 3) have an approximate 1σ error radius of less than 1500 m, while accuracy of the auxiliary classes (LC 0, A, B or Z) is poorer and highly variable (http://www.argos-system.org/html/system/faq_en.html). See electronic supplementary material for details on the algorithm we used to remove improbable auxiliary locations.

### (d) Analysis of track speed

Each reporting duty cycle lasted for 6–10 hours and produced a series of 2–10 filtered locations separated by 12–195 min. The resulting filtered duty cycles ranged from 1.5–9.2 hours. We calculated the track speed during each in-flight duty cycle as the sum of the orthodrome (great circle) distances between the bird's consecutive filtered locations divided by the total time elapsed during that duty cycle. Track speed is a function of both air speed of the bird and wind speed (Shamoun-Baranes *et al.* 2006); it provides a minimum estimate of the ground speed of the bird (actual distance travelled divided by time), since it assumes a direct flight path between reported locations. Because wind speed varied significantly along the flight path, we modelled track speed of godwits as a function of distance tracked from their departure site in Alaska, including both linear and quadratic terms for distance in a general linear model (PROC GLM; SAS Institute, Inc. 2004). We used this model to investigate the possibility of birds having stopped on any land without having been detected (electronic supplementary material).

### (e) Analysis of pressure and winds

Because our sample consisted of nine birds followed over 2 years, we combined years to assess the role of winds in the departure decisions of godwits. Thus, over the period 30 August–7 October, we compared weather conditions on days when flights commenced with intervening days when departures were not observed (acknowledging that godwits may have departed during intervening days but were undetected by our small sample of birds). Specifically, we used data from the NCEP/NCAR 40-year reanalysis project (Kalnay *et al.* 1996) to compare maps of sea-level pressure (SLP) averaged over departure days (*n*=8) with SLP averaged for all intervening days (*n*=55) to highlight broad-scale differences in the prevailing pressure-induced wind fields.

Finally, to ascertain whether godwits selected departure days that afforded advantageous wind conditions, we used two sources of data. At the ‘local’ scale, we interrogated outputs from the NASA GEOS-5 model (MAP Program 2007) to know how winds at departure correlated with departure direction. To depict conditions when birds were probably in a true migratory mode, we examined track directions of godwits relative to wind directions, both measured at a distance of 100 km from the departure site along the departure track. We calculated mean directions of their departure tracks and associated winds, calculated the mean vector length *r* (which can vary from 0 to 1) as a measure of directional concentration and tested each set for departure from randomness using the Rayleigh goodness-of-fit test (Batschelet 1981). Then, we tested the strength of the circular–circular correlation (*r*_cc_) between godwit and wind direction using the rank correlation procedure (Batschelet 1981). We do not know at what altitude godwits migrate and indeed it is unlikely that they migrate at a constant altitude, given the wind regimes they pass through on their flights. It is beyond the scope of this effort to present an analysis of winds at various heights; so we selected winds at 850 mb geopotential height as a proxy for winds used at departure. This was based on heights at which other shorebirds have been reported migrating (Green 2003) and knowledge that wind direction (but not necessarily speed) remains relatively constant between near-surface and 500 mb heights (Hanson *et al.* 1992).

For a regional perspective, we compared 850 mb wind speed and direction for days when godwit flights commenced with intervening days when departures were not observed, both relative to a 29-year climatology (1968–1996) of wind conditions over the northeast Pacific for the same calendar days (i.e. vector wind anomalies). Data for this analysis were provided by the NOAA/ESRL Physical Sciences Division, Boulder, CO (see http://www.cdc.noaa.gov/). Data on wind and climatic conditions when birds were in Melanesia and western Polynesia were derived from Kalnay *et al.* (1996), NOAA Climate Prediction Center (see http://www.cpc.noaa.gov/products/analysis_monitoring/enso_advisory) and Fiji Meteorological Service.

## 3. Results

### (a) Direct flights across the Pacific

**Figure 1:**
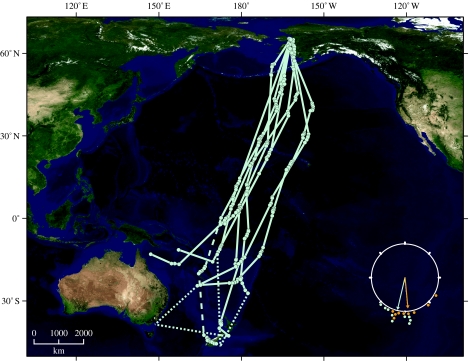
Southward flight tracks of nine bar-tailed godwits fitted with satellite transmitters (PTTs) during 2006 and 2007. Circles denote Argos locations collected during 6–8-hour intervals, and solid lines show interpolated 24–36-hour tracks between the PTT-reporting periods (see §2). Dotted lines are extensions of tracks between the last report of a PTT from a bird in flight and a confirmed sighting elsewhere of that bird. The dashed line represents the portion of flight following a confirmed stopover by a bird. Tracks are plotted on a Blue Marble image, geographic (Plate Carrée) projection (Stöckli *et al.* 2005). Inset shows individual track directions of nine PTT-tagged godwits departing on southward migration from Alaska (light blue circles) relative to directions towards which wind was blowing at 850 mb geopotential height (approx. 1500 m) during departures (orange circles). Arrows show mean direction of departing godwits (193°, light blue) and associated winds (174°, orange); length of arrows indicates strength of directionality (*r*=0.95, godwits; *r*=0.90, wind).

Nine satellite-tagged bar-tailed godwits departed Alaska between 30 August and 7 October with their initial tracks strongly concentrated (*r*=0.95, *Z*=8.19, *p*<0.001) in a southerly direction (193°; figure 1). The birds continued on this general heading across the entire central Pacific Ocean along a relatively narrow corridor (less than 1800 km wide, 10–15% of the width of the Pacific between the tropics of Cancer and Capricorn). Tracking distances from each bird's last reported location in Alaska to its first known landfall (or last reported location) ranged from 8117 to 11 680 km (10 153±1043 s.d.) for seven females with surgically implanted PTTs, and from 7008 to 7390 km for the two males with externally attached PTTs (table 1). Duration of these flights ranged from 6.0 to 9.4 days (7.8±1.3 s.d.) for birds with implants and from 5.0 to 6.6 days for birds with externally attached PTTs.

**Figure 2:**
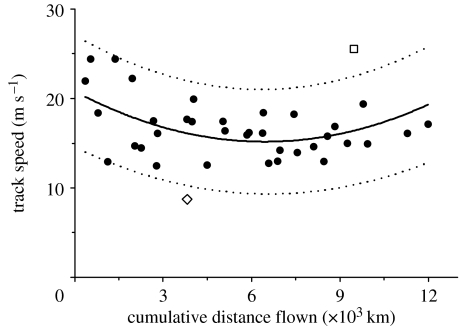
Average track speed (m s^−1^) of bar-tailed godwits during transmitter-reporting duty cycles (1.5 to 9.2-hour duration) relative to the distance tracked from Alaska (km). Track speed varied as a quadratic function of distance from departure site (*p*=0.04, *r*^2^=0.17) and reflected latitudinal differences in wind speed. Solid curve shows best-fit regression and dotted curves show 90% prediction intervals. Godwit H4 (diamond) encountered extremely strong headwinds north of Hawaii; godwit Z7 (square) was assisted by moderate tailwinds near Fiji. Track speeds of all godwits during other duty cycles (black circles) fell within 90% prediction intervals.

One female (E7) flew directly to the non-breeding area in New Zealand in 8.1 days along an overwater track encompassing 11 680 km. A second female was tracked for 6.5 days and 9621 km on a similar direct flight until her transmitter stopped reporting 1500 km offshore, but she was later observed in New Zealand and identified by her coded leg flag (Z7). Five birds were known to have landed short of reaching New Zealand: two on the Gilbert Islands (more than 7000 km), two on New Caledonia (more than 10 000 km) and one on Papua New Guinea (more than 10 000 km); two of these were subsequently observed in New Zealand after transmitters had stopped reporting. Two other individuals had flown more than 8000 km and more than 10 000 km before transmitters had stopped reporting near the Solomon Islands; the first of these was subsequently seen in New Zealand. A tenth bird whose transmitter had failed before departing Alaska was subsequently seen in New Zealand. Track speeds for the nine godwits on southward flights averaged 16.7±0.6 m s^−1^ (s.e.; range 8.7–25.5) during 37 PTT-reporting duty cycles (range 1.5–9.2 hours; mean 6.1±0.3 s.e.; 2–10 locations during each cycle). Track speed varied as a quadratic function of distance tracked from the departure site in Alaska (*p*=0.04, *F*=3.57, d.f.=2, 34), with the highest speeds generally just after departure and minimum speeds midway through the flights near equatorial latitudes (figure 2). Based on the analysis of these track speeds, only four of the godwits could have stopped on land (on six total possible occasions) during a duty cycle without being detected (table S1, electronic supplementary material). Possible stops could have been in Alaska near the beginning of the flight (within 600 km of the departure site; *n*=2), or in the South Pacific after birds had already flown more than 7000 km (*n*=4). Stopovers could only have lasted 12–67 min, even assuming extremely high flight speeds and a direct course to and from the nearest land between two known overwater locations. The much greater elapsed times (38.6±13.7 hours, range 25.1–93.8, *n*=31) between PTT-transmitting duty cycles did not allow us to evaluate robustly the potential for undetected stopovers on those long-flight segments. We could not preclude short stopovers for three other godwits whose initial flight tracks crossed over the Alaska Peninsula or Aleutian Islands between duty cycles (less than 600 km from departure). Beyond Alaska, flight tracks of six godwits passed near or through the Hawaiian Islands between duty cycles, approximately 4000 km en route. Tracks of the remaining three godwits did not come close to any land until they neared Howland Island (minimum of 6861 km), Tabiteuea (Gilbert Islands; 7023 km), and Nukufetau (Tuvalu; 7847 km). There was no evidence of a decrease in track speed (which would have suggested a stopover) during any of these long-flight segments for any godwit.

### (b) Winds and onset of migration

**Figure 3:**
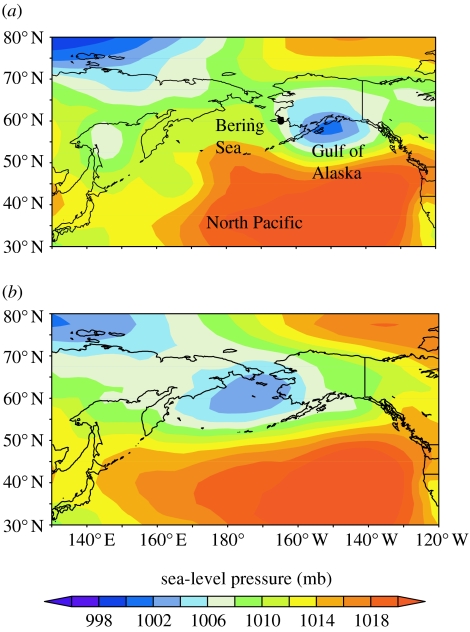
Mean SLP (mb) across the North Pacific Ocean during staging and departure of bar-tailed godwits. (*a*) Averaged for 8 days when godwits departed Alaska. (*b*) Averaged for 55 days during intervening periods when no tagged birds departed. Areas of low pressure (blue) are associated with storm centres and strong cyclonic (anticlockwise) winds in the region. Filled circle (*a*) denotes the site from which all godwits departed.

Godwits timed their departures from Alaska to coincide with specific weather systems that produced tailwinds favourable for southward flight. Wind speed 100 km en route along each godwit's departure track ranged from 3.0 to 17.5 m s^−1^ and averaged 8.3±6.1 m s^−1^ (*n*=9) at 850 mb geopotential height (approx. 1500 m). Wind directions were strongly concentrated southward (174°; *r*=0.90, *Z*=7.33, *p*<0.001; figure 1 inset) and highly correlated with the departure-track directions of individual godwits (*r*_cc_=0.88, *p*<0.001; figure S1, electronic supplementary material), suggesting a fair degree of wind assistance after take-off. The NCEP/NCAR reanalysis dataset showed that regional SLP and 850 mb winds differed considerably between days when tagged birds departed and the intervening days when tagged birds did not leave. On the 8 days on which tagged birds departed, SLP was markedly depressed over the north Gulf of Alaska, east of where godwits departed (figure 3*a*). On days (*n*=55) during which no tagged godwits departed, broad low pressure occurred over the central Bering Sea, west of where godwits departed (figure 3*b*).

## 4. Discussion

### (a) A new and extreme vertebrate model system

These non-stop flights of bar-tailed godwits, of more than 10 000 km and 9 days across the central Pacific Ocean, establish new extremes for vertebrate performance (Ashcroft 2000). Maintaining an estimated metabolic rate of 8–10 times basal metabolic rate (Pennycuick 1998; Ashcroft 2000; Gill *et al.* 2005) for more than 9 days represents a combination of metabolic intensity and duration that is unprecedented in the current literature on animal energetics (Peterson *et al.* 1990; Hammond & Diamond 1997). Furthermore, we know that the bodies of these birds undergo substantial in-flight modification beyond the loss of fat stores (Battley *et al.* 2000) and that fuel depletion may impinge on such physiological processes as metabolic substrate use, activity levels and sleep (Dewasmes *et al.* 1984, 1989; Robin *et al.* 1987; Piersma & Poot 1993). Thus, these findings raise several obvious lines of scientific inquiry into how these birds, over the length of the flight and upon arrival, manage to combine such high levels of exercise (Kvist *et al.* 2001), undergo such drastic concurrent phenotypic change (Piersma & Drent 2003), and deal with potential dehydration (Landys *et al.* 2000; Klaassen 2004; but see Schmaljohann *et al.* 2008) and sleep deprivation (Rattenborg *et al.* 2004)—all while carrying instrumentation that probably directly or indirectly influences physiological processes (Gessaman *et al.* 1991; Kvist *et al.* 2001; Piersma 2007). The bar-tailed godwit thus offers not only an exciting and tractable model system of extreme physiological performance among vertebrates, but also highlights our ignorance about how godwits (and birds in general) navigate across oceanic landscapes without landmarks (Williams & Williams 1999) and across equatorial zones where magnetic compasses cannot be used (Sandberg & Holmquist 1998).

### (b) Functional considerations: a corridor rather than a barrier

Why do godwits undertake such endurance flights and follow a transoceanic rather than a continental route? Following the coast of Asia is a well-established behaviour since godwits do so during northward migration (Barter & Wang 1990; Wilson *et al.* 2007). But clearly the central Pacific Ocean is a barrier to godwits only in the sense that there are very few sites at which they can stop and refuel, especially in the northern region. For songbirds migrating across the Gulf of Mexico, a trans-gulf route is thought to be favoured over a circum-gulf strategy owing to maximized fitness in terms of flight energetics, time en route and predation danger (Gauthreaux 1999), factors also cited for optimizing timing and routes of migration of other species (Alerstam & Lindström 1990; Hedenström & Alerstam 1997; Lind & Cresswell 2006; Hedenström 2008). Mortality during migration may be much higher than during other parts of the annual cycle (Sillett & Holmes 2002), so selection should favour behaviours that minimize risks of mortality during the migration period, including time spent accumulating the fuel resources needed for migration (Hedenström 2008).

Long-distance flights require extra energy to transport the heavy fuel loads, so detours that allow a series of shorter flights with smaller fuel loads may be advantageous in terms of minimizing energy used during transport (Alerstam 2001). Juvenile bar-tailed godwits departing Alaska on southward migration had lipid loads that were 55 per cent of their total body mass (Piersma & Gill 1998). If one assumes that godwits undertake a single transoceanic flight of 10 700 km with a relative fuel load of 1.2, one can estimate the maximum distance that godwits could detour around the barrier using the same amount of energy by carrying smaller fuel loads and refuelling at stopovers (assuming fat loads affect both parasite and induced drag during flight; eqn 4*a* in Alerstam 2001). For example, given the geometry of the coast of Asia, a single stop would require two flight steps totalling at least 15 500 km and involving a detour of 4800 km, 45 per cent more than the distance of the non-stop trans-Pacific flight. Using the same total transport energy as the trans-Pacific route but reducing the fuel load to that needed for a two-step migration would allow a maximum detour of only 29 per cent; thus the transoceanic route would clearly be more energy-efficient. Only by using five to eight flight steps (or more), which would allow for detours of up to 56–65%, could godwits use less energy to complete the total resulting flight of approximately 16 600 km (actual 55% detour); in these cases, a continental route would be predicted to be slightly more energy-efficient, in terms of migratory transport costs, than a single trans-Pacific flight. If fat depots affect only induced drag and not parasite drag during flight (eqn 4*b* in Alerstam 2001), then the transoceanic route would be more energy efficient than any of these continental routes (since two to eight flight steps would allow maximum detours of only 20–42%). Similarly, strong wind assistance at the onset of the trans-Pacific route, if unmatched along an alternative continental route, could outweigh efficiencies gained by carrying smaller fuel loads between stopover sites.

Regardless of the energy costs for transport, a single transoceanic flight probably minimizes overall time and total energy cost of migration, both of which depend on the efficiency of fuel acquisition for each step of the migration (Hedenström & Alerstam 1997). Any flight requiring extreme amounts of fuel must originate at a site where food resources are plentiful and can be acquired without other appreciable costs, including heightened exposure to predation associated with increased foraging time and fuel deposition (Houston 1998; Lank *et al.* 2003; Ydenberg *et al.* 2007). The intertidal infauna of the central and southern Yukon–Kuskokwim Delta is among the richest in the world in terms of biomass, and has been linked directly to the high intake rates of food by bar-tailed godwits prior to migration (A. Dekinga 2007, personal communication). In addition, the delta's Kuskokwim Shoals, which is the principal autumn staging site for godwits (McCaffery & Gill 2001; Gill *et al.* 2005), appears to support fewer avian predators compared with other godwit staging areas in Alaska (R. E. Gill Jr & B. McCaffery 1976–2008, unpublished data). Hence, the Kuskokwim Shoals affords a departure hub for godwits that is both rich in food and low in predation risk.

The flight corridor across the Pacific is essentially devoid of avian predators capable of taking godwits (White 1994). The peregrine falcon (*Falco peregrinus*), though recorded from most archipelagos in Oceania and a known predator of godwits elsewhere (McCaffery & Gill 2001), would be of little concern to godwits during their non-stop, largely open ocean flight since peregrines could neither consume a godwit while in flight nor land on the ocean to consume its prey. By contrast, the alternative southward migration corridor along the coast of Asia supports several species of avian predators that are capable of taking godwits (White 1994). However, New Zealand, the southern terminus of migration for most of the *baueri* population of godwits, has never been known to support any indigenous or exotic species that are considered serious predators of godwits (D. S. Melville 2008, personal communication). Thus, predation risk is minimized during most of the annual cycle, from when birds move to the staging grounds in Alaska in July until they depart New Zealand on northward migration in March.

The trans-Pacific route also provides a corridor probably free of pathogens and parasites. Long-distance migratory birds that use numerous stopovers are exposed to a diverse pathogen fauna (Piersma 1997, 2007; Mendes *et al.* 2006). Birds engaged in endurance flights presumably become energetically stressed; under such conditions, immune function could become suppressed (Apanius 1998; Råberg *et al.* 1998; Norris & Evans 2000; Owen & Moore 2006, but see Hasselquist *et al.* 2007). If godwits flew along a continental route, the greater distance would mandate at least one stopover to refuel, probably requiring several weeks' duration (cf. northbound flight; Wilson *et al.* 2007). Any immunosuppression associated with their long-distance flights could render them more susceptible to infection at stopover sites. By flying non-stop, godwits minimize their risks to novel pathogens and parasites (cf. Møller & Erritzøe 1998), and the costs of activating (Schmid-Hempel 2003) and maintaining their immune system may be reallocated into flight costs.

The trans-Pacific route does provide potential stopover areas, though godwits rarely use any except near the terminus of the migration corridor. Once tagged godwits departed the Yukon Delta staging grounds, they all passed over the Alaska Peninsula or Aleutian Islands and could have stopped there, a distance of only 500–700 km from the staging grounds. Unless a bird is seriously incapacitated, however, no advantage is accrued by stopping at this stage of the migration, especially since this is when flight costs are most commonly offset by favourable tailwinds. Once south of Alaska, godwits must cross 3600–4200 km of open ocean before reaching the Hawaiian Archipelago. Godwits crossing the archipelago either did so far from land or, if near land, would have needed to fly at implausible speeds (30–40 m s^−1^) to reach land and then return to their documented flight path. In that approximately 100 000 godwits pass over the archipelago each autumn (Gill *et al.* 2005), the paucity of sightings from the region—only 40 individuals recorded over a 35-year period (1964–1998; R. Pyle 2004, personal communication)—corroborates the godwits' propensity to fly non-stop over the Hawaiian Archipelago.

Farther south, godwits are rarely recorded anywhere in the central equatorial Pacific (Clapp & Sibley 1967; Gill *et al.* 2005). The two of our nine tagged godwits that stopped in the Gilbert Islands were the smallest individuals and the only two whose PTTs were externally mounted. We suspect that the external transmitters, either as initially mounted or after shifting owing to body mass changes, may have affected flight performance, primarily through induced drag (e.g. Obrecht *et al.* 1988; Culik *et al.* 1994). Once birds have passed through the equatorial latitudes, where winds are generally benign, they enter the realm of the southeast trades and austral westerlies, which typically generate direct or quartering tailwinds along their flight paths (Gill *et al.* 2005; figure 2). The sporadic occurrence of godwits during autumn in southern Melanesia, some 7500–9500 km from Alaska, may be evidence of a ‘fallback’ option should birds face adverse conditions within this region. One tagged godwit (H4) abruptly turned west and landed in New Caledonia instead of continuing towards New Zealand into 5–10 m s^−1^ quartering headwinds; this was after she had already battled through a cyclone with 10–15 m s^−1^ headwinds north of Hawaii (figure 2). Three other tagged godwits abruptly altered their flight trajectories and stopped in Melanesia (E5, E8 and Z0; table 1) when they encountered adverse winds associated with a strong, developing La Niña event (*sensu* Terry 2007) that brought record rainfall to the Fiji Basin (S. McGree 2008, personal communication) and an increase in southeast equatorial trades approximately 4 m s^−1^ faster than the 30-year average for this period (http://www.imarpe.gob.pe/tsm/Enso/Inicio/enso_evolution-status-09oct2007.ppt). Birds engaging in extreme endurance flights may be acutely aware of how much fuel they carry at any given time, especially near the end of such flights, and, depending on weather, opt to terminate their flights—in these cases before committing to the final leg across 1600 km of open ocean to reach New Zealand or Queensland. At the same time, we do not know how godwits may have compensated, if at all, for the fuel load needed to carry the added mass of the PTTs.

We find it unlikely that godwits would stop en route unless forced to by diminishing fuel or adverse weather conditions. Birds would probably have to reconstitute their digestive machinery if they stop to eat (Piersma & Gill 1998; Battley *et al.* 2000; Landys-Ciannelli *et al.* 2003), and long-haul migrants appear physiologically adapted to minimize water loss (Landys *et al.* 2000; Liechti *et al.* 2000; cf. Klaassen 2004). Stopping to drink would require added energy expenditure to climb back to flight altitude, since the rate of fuel burn is the greatest during the launch and climb phases of the flight (Landys *et al.* 2000). We assume that Pacific godwits mostly migrate at altitudes similar to or higher than those of East Atlantic flyway godwits (2000–5000 m; Landys *et al.* 2000), given the numerous latitudinally defined wind regimes the Pacific birds encounter on their transoceanic flights. All evidence suggests that if godwits are forced to stop en route, they do so for prolonged periods (days or weeks as opposed to hours or minutes).

Winds are clearly an integral aspect of the godwits' transoceanic flight south from Alaska, but would birds similarly avail of favourable winds to launch flights to Asia if a continental route were followed? The cyclones on which godwits depart develop in the northwest Pacific and move east towards the Gulf of Alaska (Brower *et al.* 1988). The flight of at least one species of bird migrating from Alaska to Asia during autumn is sometimes assisted by winds at departure (*Branta bernicla nigricans* to Japan; Derksen *et al.* 1996). However, there is no steering trough off Asia, as there is in the Bering Sea and Gulf of Alaska, which would regularly promote extended southward flow along the coast of Asia (G. Hufford 2008, personal communication). Without such atmospheric structure, there is much less ‘predictability’ to promote a wind-aided continental migration strategy for godwits.

Despite the advantages of a trans-Pacific route during southward migration, godwits do follow a continental route during northward migration. They probably take this longer route north because (i) ‘overloading’ at a final coastal stopover site before reaching the breeding grounds could give godwits an advantage during breeding (cf. Alerstam & Hedenström 1998; Alerstam 2001; Morrison *et al.* 2005) and (ii) there are no landing sites for the last 4000 km of the trans-Pacific flight path should the birds run out of fuel heading north.

### (c) Wind assistance and potential climate change effects

Favourable winds—both at departure and en route—can confer considerable savings in time and energy and many species of long-distance migrants incorporate winds into their migration strategies (Alerstam 1990; Landys *et al.* 2000; Liechti 2006). However, for winds to be a selective factor in the evolution of a strategy, they must be measurable and predictable. Departures of godwits from Alaska were associated with tailwinds (cf. Gill *et al.* 2005) generated by the Aleutian low-pressure centre, arguably the most dynamic of the semi-permanent, large-scale synoptic features driving Northern Hemisphere circulation (Wilson & Overland 1986). During the godwit departure period (late August to early November), between two and five cyclones track into the Gulf of Alaska each month that generate winds favourable for southward departure (Brower *et al.* 1988). Such predictability would enable a wind-assisted departure strategy to evolve. We are uncertain, however, about the antiquity of this population of godwits and how their migration strategy may have been influenced by palaeoclimatic shifts in storm tracks across the Pacific. The godwits' current breeding and staging areas did remain ice-free during Pleistocene glaciations (Hamilton *et al.* 1986), but palaeoclimate modelling suggests that the Aleutian low-pressure centre intensified and storm tracks probably shifted during the Last Glacial Maximum (Heusser *et al.* 1985; Kaspar *et al.* 2007; Yanase & Abe-Ouchi 2007).

There is mounting evidence that global climate change is currently affecting migratory birds, principally through changes to their habitat and migrational timing (Cotton 2003; MacMynowski & Root 2007; Meltofte *et al.* 2007). Much less clear is what effect the changes in atmospheric circulation may have on wind-selected migration. Most coupled climate-model ensembles predict a significant increase in either the number or intensity of winter cyclones in the mid-latitude North Pacific (with unknown ramifications for the autumn migration period), and some models predict a northward shift in the prevailing storm track (McCabe *et al.* 2001; Yin 2005; Bengtsson *et al.* 2006; Salathé 2006; Pinto *et al.* 2007; Raible 2007; Loptien *et al.* 2008). Changes in the timing, intensity or spatial distribution of cyclonic activity in the North Pacific could have implications for the godwits' present-day migration strategy.

Some projected climate changes would tend to favour the godwits' southward migration by providing more opportunities for wind-assisted departures (increased frequency of cyclones) or enhanced wind assistance (more intense cyclones). By contrast, a northward shift in the principal autumn storm track could impair favourable wind regimes by introducing persistent blocking ridges and strong headwinds at the godwit staging areas, conditions that are currently rare during the August–October period (Wilson & Overland 1986). Such a shift did occur during September 2007, when the east Pacific high-pressure ridge was unusually strong and, for almost a three-week period, deflected cyclones into the Bering Sea from their normal trajectory into the Gulf of Alaska and produced strong headwinds for departing godwits (G. Hufford 2008, personal communication). Not surprisingly, upon breakdown of the 2007 blocking ridge, two of three tagged godwits that remained in Alaska departed on the first storm to track into the Gulf of Alaska. If storm tracks were to shift permanently northward, it is not likely that godwits could similarly shift departure sites, since staging areas are determined by more persistent geomorphological features (riverine deltas).

The central Pacific Ocean provides a unique migration corridor that allows bar-tailed godwits not only to minimize the time and total energy allocated to migration but also to minimize risk of mortality from predators, parasites and pathogens. Use of this corridor is facilitated by salubrious conditions at the main departure hub on the Yukon–Kuskokwim Delta, which hosts a remarkably biomass-rich invertebrate fauna and relatively scarce predators. Few predators at the principal staging area and lack of predators and pathogens during the non-stop flight allow godwits to maximize fuel deposition required for endurance flight. Large-scale weather systems track predictably across the departure region and provide significant wind assistance during early migration. Islands in the south Pacific may provide fallback landing sites should godwits encounter unfavourable conditions before reaching the terminus of their flight. What points remain unclear are how much tolerance individual godwits have in their migration strategies and how survival rates might be affected if weather conditions at their departure hub or along the corridor should change.

## Figures and Tables

**Table 1:** Histories of bar-tailed godwits fitted with satellite transmitters and tracked on southward migration from Alaska in 2006 and 2007.

				non-stop track		
					
bird ID^a^	sex	PTT type^b^	date departed^c^	distance (km)	time (days)	first known landfall or last signal received
H6^d^	male	s	23 Sep 2006	7008	5.0	Tarawa, Gilbert Islands
Z3	male	s	21 Sep 2006	7390	6.6	Nonouti, Gilbert Islands
BØ^e^	female	i	30 Aug 2006	8117	6.0	open ocean, 250 km NE Anuta, Solomon Islands
Z7^f^	female	i	31 Aug 2006	9621	6.5	open ocean, 1500 km NNE NZ
E8	female	i	7 Oct 2007	10 026	9.4	Pavuvu, Solomon Islands
E5^g^	female	i	21 Sep 2007	10 080	7.3	Pouebo, New Caledonia
ZØ	female	i	23 Sep 2007	10 607	8.1	Puro Bay, Papua New Guinea
H4	female	i	10 Sep 2006	10 940	9.2	Ouvêa, New Caledonia
E7	female	i	30 Aug 2007	11 680	8.1	Piako River Mouth, North Island, NZ

a Each bird received a unique alphanumeric-coded leg flag placed on the tibiotarsus.

b Surgically implanted battery-powered PTT (i); externally attached solar-powered PTT (s).

c All the birds departed from the south Yukon–Kuskokwim Delta, Alaska.

d After stopping in Tarawa, H6 resumed migration on 2 November and was tracked to within 120 km of Farewell Spit, South Island, New Zealand.

e Godwit BØ was subsequently seen on Farewell Spit, New Zealand, in February 2007.

f Godwit Z7 was subsequently seen on Farewell Spit, New Zealand, in December 2006.

g Godwit E5 was subsequently seen in New South Wales, Australia, on 8 December 2007 and then at Miranda, North Island, New Zealand, on 14 March 2008.

## Supplementary Material

Additional materials, methods, tables and figures Filtering of location data; Analysis of track speed; Figure S1; Table S1
