# Supplementary material for: Extreme endurance flights by landbirds crossing the Pacific Ocean: ecological corridor rather than barrier?
Source: Proc Biol Sci. 2008 Oct 29;276(1656):447–57. doi: 10.1098/rspb.2008.1142 (PMC2664343; doi:10.1098/rspb.2008.1142)
Supplement: Additional materials, methods, tables and figures — Filtering of location data; Analysis of track speed; Figure S1; Table S1 [file rspb20081142s04.doc]

**Electronic supplementary material**

Gill Jr, R. E. *et al.* 2008 Extreme endurance flights by landbirds crossing the Pacific Ocean: ecological corridor rather than barrier? *Proc. R. Soc. B* (**doi:10.1098/rspb.2008.1142**)

***Filtering of location data***

We used a systematic filtering algorithm (**http://alaska.usgs.gov/science/biology/**

**spatial/douglas.html**) to remove improbable auxiliary locations. Auxiliary locations within 10 km of a preceding or subsequent location were retained by virtue of spatial redundancy, and the remaining auxiliary locations were retained only if resultant movement rates were <33.3 m s^-1^ and the internal angles (α, in degrees) formed by preceding and subsequent vectors (of lengths *d*1 and *d*2 km) were not suspiciously acute (α > -25 + ß × ln[minimum (*d*1,*d*2)], where ß = 15). We assigned ß = 15 because it performed well for our specific tracking data both before and during migration. In our case, all of the godwit locations that were filtered during migration exceeded the rate threshold, so our somewhat liberal coefficient for the angle test (i.e., ß = 15) was inconsequential to the broad-scale migration tracks. While increasing the value for ß causes the filter to be less tolerant of angular deviations from a straight path, birds do sometimes abruptly change the direction of their migrations and we did not wish to falsely reject these changes. Criteria to filter Argos data are best established with iterative quality control and assessment, and results should be expected to vary across species and behaviours. Of the 297 total Argos locations recovered during migration, 71 were standard locations that were always retained, whereas 15 per cent of 226 auxiliary locations were filtered, leaving a total of 264 locations for analysis. Given the spatial scales considered herein, this filtering approach is suitable for establishing and analysing long-distance movement paths.

***Analysis of track speed***

We investigated the possibility of birds having stopped on any land without having been detected. For flight segments that passed close to atolls or islands, we estimated the length of time that a godwit could have possibly spent on land had it deviated from the assumed direct flight path over water. First we calculated great-circle distances to and from the closest land between adjacent filtered locations at which the bird had been reported. We then estimated the time that would have been required for the alternate flight path to land, given that the bird had maintained a track speed equivalent to the upper 90% prediction interval estimated by the general linear model (see §2) for individual birds at that distance from the departure site. Finally, we calculated the maximum layover time possible (if any) that each bird could have spent on land by subtracting the required flight time from the total elapsed time between the two known over-water locations. We consider this to be a conservative analysis, because if godwits had significant wind assistance in one direction they would most certainly have encountered significant wind resistance in the opposite direction during the short time periods (min) between their known locations.


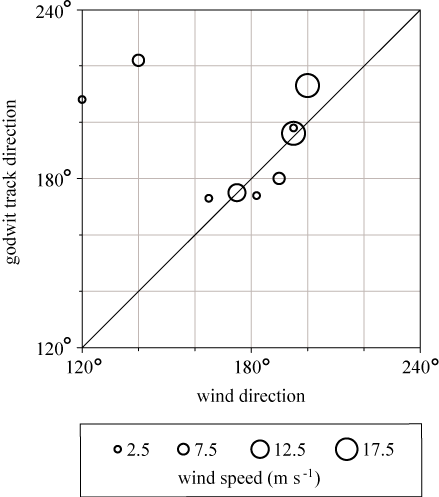


Figure S1. Track directions of nine PTT-tagged godwits departing on southward migration from Alaska relative to wind speed and direction (towards which it was blowing) at 850-mb geopotential height (~1500 m), both measured at a distance of 100 km from the departure site. Departures of seven godwits were assisted by tailwinds (exact coincidence would be along diagonal line); two birds faced light to moderate crosswinds (upper left quadrant).

**Table S1. Maximum possible stopover time for godwits that could have flown undetected to nearest land between two reported overwater locations during PTT duty cycles.**

| bird ID^a^ | land nearest to reported overwater track locations | track distance from Alaska departure site (km) | assumed ground speed (m s^-1^)^b^ | maximum stopover time (min) ^c^ |
| --- | --- | --- | --- | --- |
|  |  |  |  |  |
| E7 | Izembek Lagoon, Alaska | 538 | 25.7 | 40 |
| E7 | Otter Cove, Alaska | 635 | 25.7 | 17 |
| E7 | Cape Reinga, New Zealand | 11 292 | 24.7 | 67 |
| H4 | Nagigia, Fiji | 9 671 | 22.8 | 48 |
| H6 | Espiritu Santo, Vanuatu | 9 069 | 22.2 | 33 |
| ZØ | Abemama, Kiribati | 7 366 | 21.2 | 12 |

^a^ Each bird received a unique alphanumeric-coded leg flag placed on the tibiotarsus.

^b^ Assumed ground speed (actual distance travelled divided by time in flight) was liberally estimated as the upper 90% prediction interval of track speed for individual birds at the respective distance from the departure site (based on the general linear model of godwit track speeds measured during 37 PTT duty cycles; see Material and Methods).

^c^ Calculated as difference between actual elapsed time between two reported within-duty-cycle overwater locations and the time required to fly directly to and from the nearest land at assumed ground speed.
